# Smoking histories: a bioarchaeological approach to tobacco consumption in two skeletal populations from The Netherlands (1300-1829 CE)

**DOI:** 10.1080/00794236.2024.2355461

**Published:** 2024-05-31

**Authors:** Maia Casna, A. M. Davies-Barrett, S. A. Schrader

**Affiliations:** 1 Faculty of Archaeology, Leiden University, Leiden, The Netherlands; 2 School of Archaeology and Ancient History, University of Leicester, Leicester, UK

**Keywords:** Paleopathology, pipe-notches, dental staining, post-medieval

## Abstract

SUMMARY: Over the past decade, the history of tobacco’s introduction to Europe and its societal impact has been extensively studied, resulting in prevailing narratives about its adoption and consumption. In the Netherlands, historical records generally concur that: (I) tobacco rose in popularity among all socioeconomic classes between 1590 and 1630 CE; and (II) it spread throughout the Country as a male habit. However, the presence and consumption of tobacco have exhibited profound variations across diverse societies throughout history, manifesting dissimilar patterns of employment and significance over varying temporal and spatial dimensions. By analysing a sample of 351 human skeletons dating from 1300 to 1829 CE, the present study challenges the limited historical narratives presented above and emphasizes the diverse contextual factors that influenced tobacco’s prevalence in two different Dutch centres. Our results suggest that in certain areas of the Netherlands tobacco was likely present and widely consumed well before 1630 CE, while also highlighting overall substantial female participation in the practice. Furthermore, our analysis hints at the possibility of divergent methods of tobacco consumption between sexes, suggesting that the historical narrative of tobacco as solely a male habit may warrant reconsideration. Overall, our study contributes to a deeper understanding of the complex history of tobacco in the Netherlands, shedding light on historical trends and cultural practices.

## INTRODUCTION

Tobacco was first introduced to Europe by the Spanish at the end the sixteenth century, initially as a remedy for various ailments (e.g. headaches, toothaches, intestinal worms), and later as an intoxicant (Fraga 2010; Goodman 2005). Unlike other trade substances in the Netherlands, such as coffee and sugar, the spread of tobacco did not originate from the higher socioeconomic classes; instead, it started with seamen and lower-class individuals who would gather in port inns and taverns to smoke after work (Snelders 2021). By 1630 CE, the habit of consuming tobacco had permeated across all strata of society, establishing itself as a significant commodity within the national and international market. According to historical sources, what truly allowed tobacco to become such a popular commodity in less than 50 years was the emergence of the pipe-maker business in 1609 CE (Brongers 1964a; Snelders 2021). Pipes likely provided a more efficient and enjoyable way to consume tobacco compared to other methods available at the time (e.g. chewing, snuffing). The act of smoking through a pipe allowed users to inhale larger quantities of tobacco, leading to increased consumption and to higher demand (Brongers 1964b). The presence of pipe fragments in most Dutch archaeological excavations dating to 1650 CE and onwards attest to how, in the post-medieval period, smoking had become an important part of Dutch culture, regardless of socioeconomic status or geographical location. In a recent study by Inskip et al. (2023) on the impact of tobacco on the oral health of a post-medieval rural Dutch population, it was observed that at least 71.7% of male individuals showed dental evidence of smoking (e.g. pipe notches), suggesting the consumption of tobacco was not exclusive to larger urban centres (see also Bartholdy et al. 2024).

Despite its popularity across all socioeconomic classes, just a few years after its introduction tobacco seems to have become exclusively associated with men (Brongers 1964b). While the habit of smoking was largely tolerated and, in some contexts, encouraged among men, according to many historical sources tobacco use was considered distasteful and immoral for women, often linked to overt sexuality, prostitution, and unappealing attributes (McShane 2021; Snelders 2021). While Brongers (1964b) argues that Dutch women (particularly those from rural areas) have consistently engaged in some degree of smoking, the predominant belief that tobacco use was mainly associated with men prevails to this day, often coupled with limited historical documentation specifically focused on women (McShane 2021). The resulting bias appears to be particularly evident in bioarchaeological studies conducted on Dutch populations where, up to now, most published research on past tobacco consumption only encompasses male individuals (e.g. Bartholdy et al. 2024; Inskip et al. 2023).

To address these biases in historical pre-conceptions about tobacco consumption in Europe, we analysed dental evidence of tobacco usage in two skeletal populations from the Netherlands, dating to 1300-1829 CE.

### BIOARCHAEOLOGICAL APPROACHES TO TOBACCO CONSUMPTION

Despite the direct insights provided by analysis of archaeological human skeletal remains into the identification of tobacco consumption at the level of the individual, the presence of lesions associated with smoking has been, to date, only scarcely addressed in bioarchaeology. Some biomolecular studies have explored the presence of tobacco through the examination of human dental calculus or metabolomic data (e.g. Badillo-Sanchez et al. 2023; Bartholdy et al. 2024), while macroscopic, non-destructive methods have also proven to be valuable tools to address tobacco consumption, especially when dealing with larger samples. In a study conducted by Walker and Henderson (2010), which examined the impact of smoking on the health of a 19th-century skeletal population from London, the presence of either (1) pipe notches; or (2) dental staining on the inner (i.e. lingual) surface of teeth was considered as indicative of tobacco consumption. Pipe notches (here defined as semi-circular abrasions attributed to teeth clenching around clay pipes) have become a routine component of skeletal analyses today. However, only a limited number of studies have addressed their prevalence at the populational level, with reported rates ranging from 39.6% to 77.3% for populations known to have had access to tobacco (Geber and Murphy 2018; Inskip et al. 2023; Walker and Henderson 2010).

The second form of evidence (i.e. dental staining) is allegedly unaffected by the type of tobacco consumption (e.g. clay pipes, chewing) and is instead attributed to the chemical composition of tobacco itself (Kiliçarslan et al. 2022; Dalrymple et al. 2021). Because of this, it was suggested that dental staining has the potential to provide more comprehensive insights into tobacco consumption, extending beyond pipe usage (Davies-Barrett and Inskip 2024). Unfortunately, dental staining is today largely overlooked, with only a few studies addressing its presence within archaeological samples. Previous investigations into the prevalence of dental staining among archaeological skeletal populations have yielded varying results, with prevalence rates ranging from 25.9% to 48.8% (Davies-Barrett and Inskip 2024; Walker and Henderson 2010).

### HYPOTHESES

While historical studies of tobacco consumption provide plenty of valuable insights into the impact of tobacco on the health of past peoples, their limited scope and data availability often preclude a comprehensive analysis of the spread and long-term impact of tobacco use on Dutch populations across time and space. To begin addressing this knowledge gap, our study examines the presence of macroscopic evidence for tobacco consumption on human dentition (i.e. pipe notches, dental staining) in various archaeological populations from the Netherlands. Informed by the data presented by historical studies on tobacco consumption in the Netherlands, two hypotheses were formulated:

1. Following the introduction of tobacco in Europe and in the Netherlands at the end of the 16th century, its usage had become extremely popular among all socioeconomic classes by 1630 CE. We hypothesized that populations with documented access to tobacco would exhibit heightened skeletal indicators of tobacco consumption (i.e. pipe notches and dental staining) in comparison to their predecessors from earlier periods.
2. According to historical sources, men were more likely than women to consume tobacco due to the cultural norms and gender-specific expectations surrounding smoking. We hypothesized statistically significant differences in smoking evidence between male and female skeletal sex categories.

## MATERIALS

For the purpose of this study, we selected two skeletal urban populations (i.e. Arnhem and Vlissingen) with known historical backgrounds related to tobacco. The total sample comprised 351 individuals whose age-at-death was ≥20 years. Based on the available dating of the skeletal remains, each population was categorized into two distinct groups: (1) ‘pre-tobacco’, denoting periods predating the widespread adoption of tobacco among European populations; and (2) ‘post-tobacco’, indicating eras following the dissemination of tobacco and integration into common customs and practices (Table 1).

**TABLE 1:** Demographic composition of the sample included in this study.

		Sex		Age-at-death			
		Male	Female	Young adult (20-34 years)	Middle adult (35-49 years)	Old adult (50+ years)	Total
Arnhem	Pre-tobacco (1330-1650 CE)	54	50	49	34	21	104
	Post-tobacco (1650-1829 CE)	48	56	34	30	40	104
Vlissingen	Pre-tobacco (Oude Markt, 1300-1590 CE)	27	25	33	14	5	52
	Post-tobacco (Scheldekwartier, 1600-1800 CE)	45	46	59	17	15	91

### ARNHEM

**FIG. 1:**
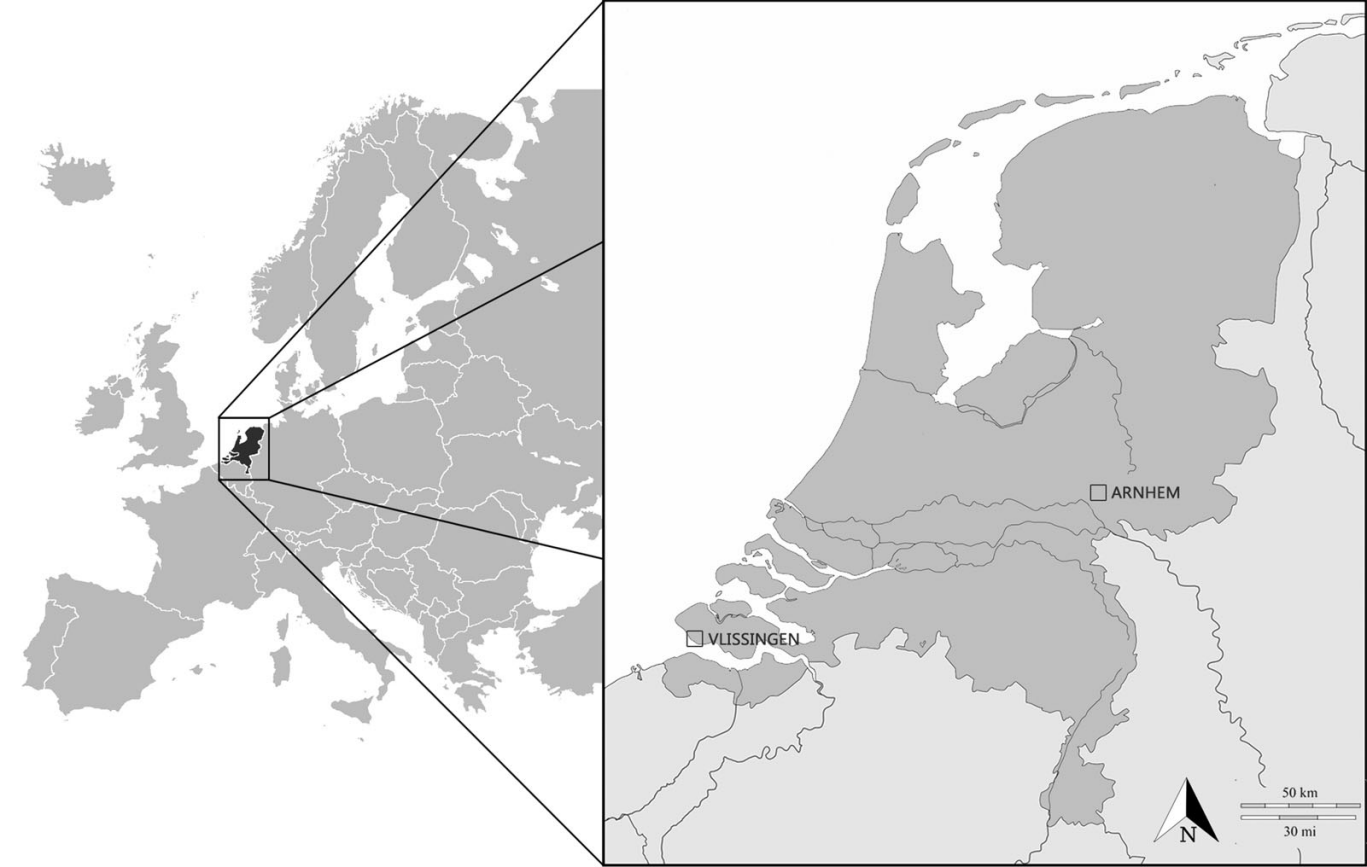
Map of The Netherlands showing the location of the sites under study.

The skeletal assemblage of Arnhem (Fig. 1) was excavated in 2017 in the courtyard of St. Eusebius’ Church and comprises approximately 350 skeletons interred between 1330-1829 CE (Zielman and Baetsen 2020). Relative and absolute dating of the graves were performed by Zielman and Baetsen (2020) by creating a Harris Matrix and a 3D model to gain insight into the chronological development of the cemetery. To establish absolute dating, bone collagen samples were extracted from the ribs of 16 individuals and subjected to 14 C analysis. The rest of the skeletal population was assigned a relative dating based on their spatial relationship to the 16 radiocarbon-dated individuals (Zielman and Baetsen 2020). For the purpose of this study, each skeleton was attributed a specific dating range that corresponds to two time windows: 1330-1650 CE (Pre-tobacco) and 1650-1829 CE (Post-tobacco), respectively.

The history of post-medieval Arnhem is intricately intertwined with tobacco. From the beginning of the eighteenth century, tobacco was one of the most important crops grown in the river area where Arnhem is located. According to historical sources, the first tobacco spinning mill in the surroundings of Arnhem was built and put into function in 1705 CE and, from there, urban production increased exponentially in less than 50 years (Roessingh 1976). By 1740 CE, about 3 million pounds of dried tobacco leaves were weighed annually in Arnhem alone (Kuiper and Lengkeek 1994). Following the increase in local agricultural production, several tobacco factories opened in Arnhem during the eighteenth century, quickly becoming the main employers for people relocating to the city in search of work (van Laar 1966). The individuals buried in the courtyard of St. Eusebius’ Church and analysed in this study are thought to be representative of the lower working class (Casna et al. 2021). Therefore it is likely that at least some of them were employed in the tobacco industry. However, because of how affordable and popular tobacco was at the time, it is also likely that most individuals from the post-tobacco sample had ample access to the commodity regardless of their employment situation (Brongers 1964b; Roessingh 1976).

### VLISSINGEN

Today, Vlissingen is a city located in the southwestern Netherlands, on the former island of Walcheren, in the province of Zeeland. With its strategic location between the Scheldt River and the North Sea, Vlissingen has been an important harbour for centuries, managing several commercial routes to South America and the Caribbean as early as the seventeenth century on behalf of the United East India Company (van Cruyningen 2012).

From 2003 to 2008, different excavations in the city harbour area uncovered a total of 844 burials dated 1300-1590 CE (Oude Markt, *n* = 716) and 1600-1800 CE (Scheldekwartier, *n* = 128), respectively (Claeys, Jaspers, and Ostkamp 2010). Whilst differences might exist between the demographic profiles of the two samples, the burials recovered from Oude Markt and Scheldekwartier are both thought to be representative of the lower socioeconomic class and, hence, to be comparable to the skeletal assemblage of Arnhem (Claeys, Jaspers, and Ostkamp 2010).

The presence of tobacco consumption in Vlissingen was partly addressed by Claeys, Jaspers, and Ostkamp (2010) in the analysis of the archaeological evidence recovered from the excavations: macroscopical evidence of smoking (i.e. pipe notches) was observed in circa 20% of the examined individuals from Scheldekwartier. The relatively small number of clay pipe fragments recovered on site and the dating of most fragments between 1740 and 1860 CE suggest that tobacco smoking became popular in Vlissingen somewhat later than in other harbour cities in the Netherlands, where clay pipes date much earlier (Claeys, Jaspers, and Ostkamp 2010). Nevertheless, historical records indicate that tobacco plantations existed in Zeeland as early as 1610, albeit for medicinal uses (Enthoven 1996).

## METHODS

Standard osteoarchaeological methods were used to estimate the sex and age-at-death of each individual. Skeletal sex was estimated based on the observation of morphological features on the skull (Buikstra and Ubelaker 1994) and os coxae (Bruzek 2002; Phenice 1969). Age estimations of adults were made by observing the morphology of the pubic symphysis (Brooks and Suchey 1990) and the auricular surface (Buckberry and Chamberlain 2002). Age-at-death categories were classified as young adult (20-34 years), middle adult (35-49 years), and old adult (50+ years) (Buikstra and Ubelaker 1994).

### EVIDENCE FOR TOBACCO CONSUMPTION

**FIG. 2:**
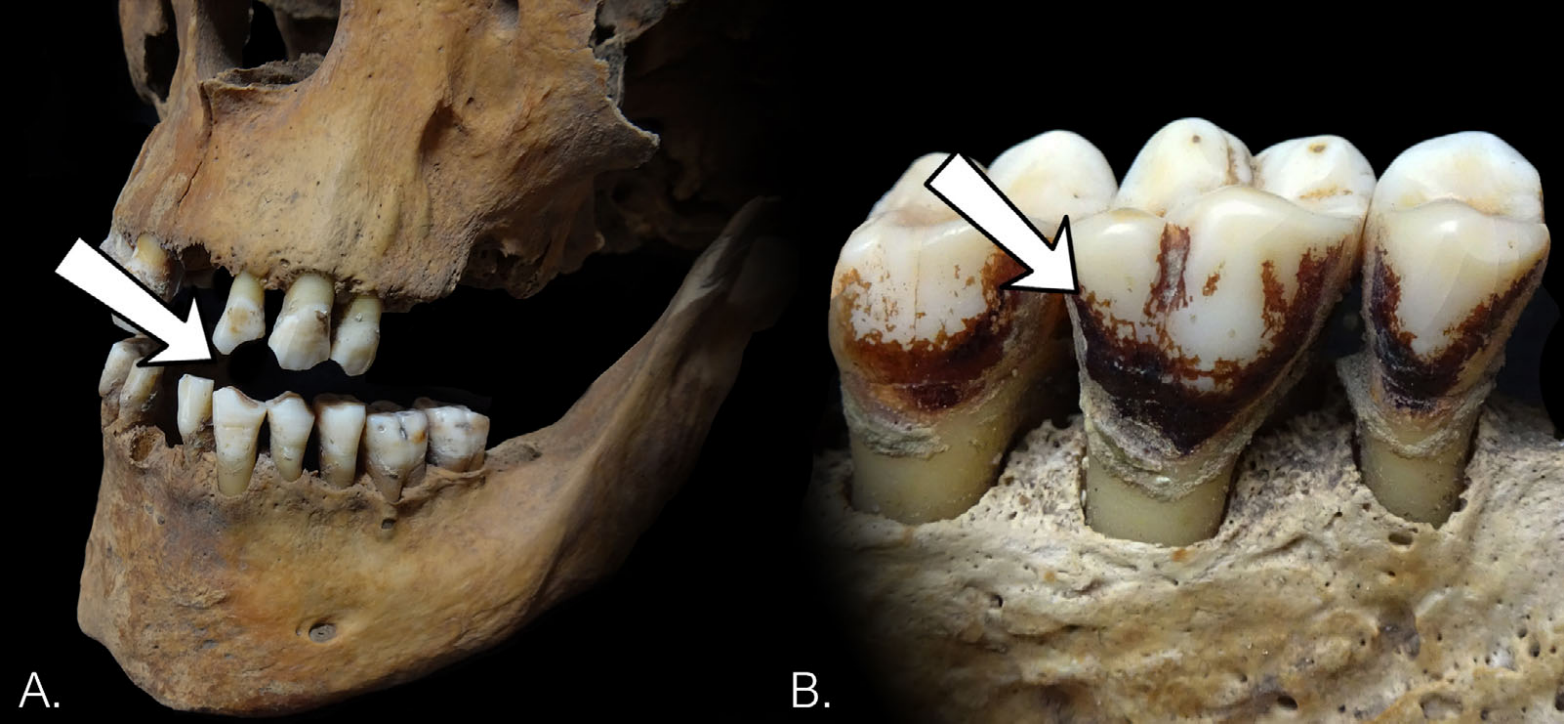
Examples of evidence for tobacco consumption considered in this study: (A) pipe notch, (B) dental staining on lingual surfaces. Photographs by M. Casna.

Assessment of tobacco consumption at the level of the individual was based on: (1) the presence of pipe notches; and (2) the presence of dental staining on the lingual surfaces of the dentition, as presented by Davies-Barrett and Inskip (2024) (Fig. 2). If the dentition was observable, the individual was marked as either ‘present’ or ‘absent’ for each type of dental change. Overall evidence of tobacco consumption was scored as ‘present’ for individuals who displayed at least one of the markers we considered indicative of tobacco use (i.e. pipe notches and dental staining). This allowed a calculation of the overall prevalence of evidence for tobacco consumption in the sample group.

#### PIPE NOTCHES

The presence of pipe notches (Fig. 2A) was assessed for all available dentition within an individual, except for the molars. Accurately determining the presence or absence of pipe notches is difficult as it requires a relatively complete set of incisors, canines, and premolars, as the absence (either postmortem or antemortem) of certain teeth could obscure the fact a pipe notch may have originally been present but is no longer observable. Thus, according to the method outlined by Davies-Barrett and Inskip (2024), the intersections between the incisors, canines, and premolars were divided into nine ‘sites’ across a single jaw, consisting of a pair of neighbouring teeth (sites: LP2-LP1, LP1-LC, LC-LI2, LI2-LI1, LI1-RI1, RI1-RI2, RI2-RC, RC-RP1, RP1-RP2)^1^. A site was only considered as observable if both neighbouring teeth were present. In order for an individual to be assessed for the presence of a pipe notch, it was required that one of each of the nine sites was accounted for in either the upper or lower jaw. An individual received a score of ‘unobservable’ if there was a lack of at least one site in both the upper and lower dentition (e.g. both the sites of the LC^1^-LP^1^ and LC_1_-LP_1_ were absent).

#### DENTAL STAINING

Dental staining is defined as the adhesion of a substance with a variable light brown to black colour to the lingual (i.e. inner) tooth surface (Davies-Barrett and Inskip 2024). According to clinical sources, dental staining results from nicotine and/or tar from tobacco permeating the enamel’s micropores, causing noticeable discoloration of the teeth surface (Dalrymple et al. 2021; Kiliçarslan et al. 2022). This process may be influenced by several factors other than tobacco throughout the life of an individual, such as coffee and tea consumption, personal oral hygiene practices, and inhalation of other forms of smoke (Ness, Rosekrans, and Welford 1977). To discern tobacco-induced stains from those originating from other sources, we observed distinctive characteristics unique to tobacco stains: a) they usually manifest as a lacquer-like coating located exclusively on the lingual surface, unlike stains from alternative substances which typically affect the entire tooth; and b) their appearance resembles that of 'cracked ink’ or a 'dry riverbed’, setting them apart with their distinct visual pattern (Davies-Barrett and Inskip 2024) (Fig. 2B).

To ensure comparability with other studies on tobacco consumption (e.g. Davies-Barrett and Inskip 2024), in this paper the presence of dental staining was assessed in individuals who had at least 25% (8 out of 32) of observable dental lingual surfaces present. Dental staining was recorded as ‘present’ for individuals where at least one tooth showed surface discoloration.

### STATISTICAL ANALYSIS

Statistical analysis was performed using SPSS for Windows, version 29.0. The significance between variables was investigated using Chi-squared tests. Two-tailed Fisher’s exact tests were utilized when the expected cell count was less than 5. To address the issue of multiple tests being run on the same samples, the Bonferroni correction method was applied to adjust the significance levels and account for familywise error. In all statistical tests, an original p-value ≤0.05 was considered to be statistically significant, with a final applied Bonferroni corrected p-value of ≤0.008 indicating a statistically significant outcome.

## RESULTS

**FIG. 3:**
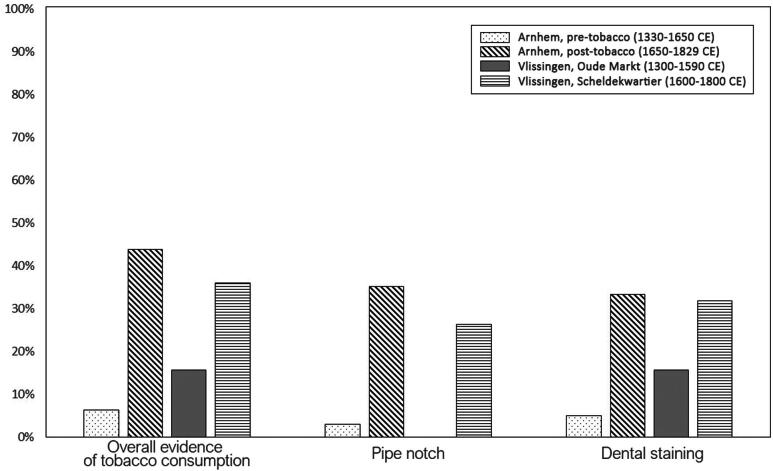
Prevalence of evidence of tobacco consumption, pipe notches, and dental staining for all samples under study (*N* = 239).

Individuals from the post-tobacco skeletal samples showed higher rates of overall evidence of tobacco consumption, pipe notches, and dental staining (Table 2; Fig. 3).

**TABLE 2:** Frequencies of observed skeletal markers for tobacco consumption.

		Pipe notch (N)	Dental staining (N)	Overall evidence of tobacco consumption (N)	Co-occurrence of pipe-notch and dental staining (N)
Arnhem	Pre-tobacco (1330-1650 CE)	2 (70)	4 (79)	5 (79)	2 (70)
	Post-tobacco (1650-1829 CE)	20 (57)	21 (63)	28 (64)	13 (56)
Vlissingen	Pre-tobacco (Oude Markt, 1300-1590 CE)	0 (22)	2 (32)	5 (32)	0 (22)
	Post-tobacco (Scheldekwartier, 1600-1800 CE)	16 (61)	20 (63)	23 (64)	13 (60)

N = total of individuals with observable feature.

Table 3 presents the results of the statistical analysis conducted to assess tobacco consumption across various time periods and among all examined skeletal populations. In the Arnhem sample, tobacco consumption was found to be significantly higher in the post-tobacco period than in the pre-tobacco one for all tobacco consumption categories (i.e. overall evidence of tobacco consumption, pipe notch, and dental staining). However, in Vlissingen, statistical variation between pre- and post-tobacco periods was observed solely concerning pipe notches.

**TABLE 3:** Statistical analysis of tobacco consumption in different time periods and skeletal populations.

Comparison	Skeletal assemblage		Total of individuals with observable feature	Total of individuals presenting feature (%)	χ2	*p*-value
Overall evidence of tobacco consumption versus time period	Arnhem	Pre-tobacco	79	5 (6.3)	27.062	**<0.001**
		Post-tobacco	64	28 (43.7)		
	Vlissingen	Pre-tobacco (Oude Markt)	32	5 (15.6)	4.261	0.039
		Post-tobacco (Scheldekwartier)	64	23 (35.9)		
Pipe notch versus time period	Arnhem	Pre-tobacco	70	2 (2.9)	22.789	**<0.001**
		Post-tobacco	57	20 (35.1)		
	Vlissingen	Pre-tobacco (Oude Markt)	22	0 (0)	n/a	**†0.005**
		Post-tobacco (Scheldekwartier)	61	16 (26.2)		
Dental staining versus time period	Arnhem	Pre-tobacco	79	4 (5.1)	19.310	**<0.001**
		Post-tobacco	63	21 (33.3)		
	Vlissingen	Pre-tobacco (Oude Markt)	32	5 (15.6)	2.844	0.092
		Post-tobacco (Scheldekwartier)	63	20 (31.7)		

†=Fisher’s Exact Test. Values in bold indicate associations statistically significant by Chi-square/Fisher’s Exact test, with Bonferroni correction significance level set at *p* = 0.008.

We examined the co-occurrence of pipe notches and dental staining within each sub-sample (i.e. Arnhem pre- and post-tobacco, as well as Vlissingen, Oude Markt, and Scheldekwartier). Statistically significant co-occurrences were observed in all sub-samples (Arnhem, pre-tobacco: *p* = 0.002; Arnhem, post-tobacco: χ^2^=11.731, *p* < 0.001; Vlissingen, Scheldekwartier: χ^2^=27.959, *p* < 0.001), except for Vlissingen and Oude Markt, where no pipe notches were observed at all.

**FIG. 4:**
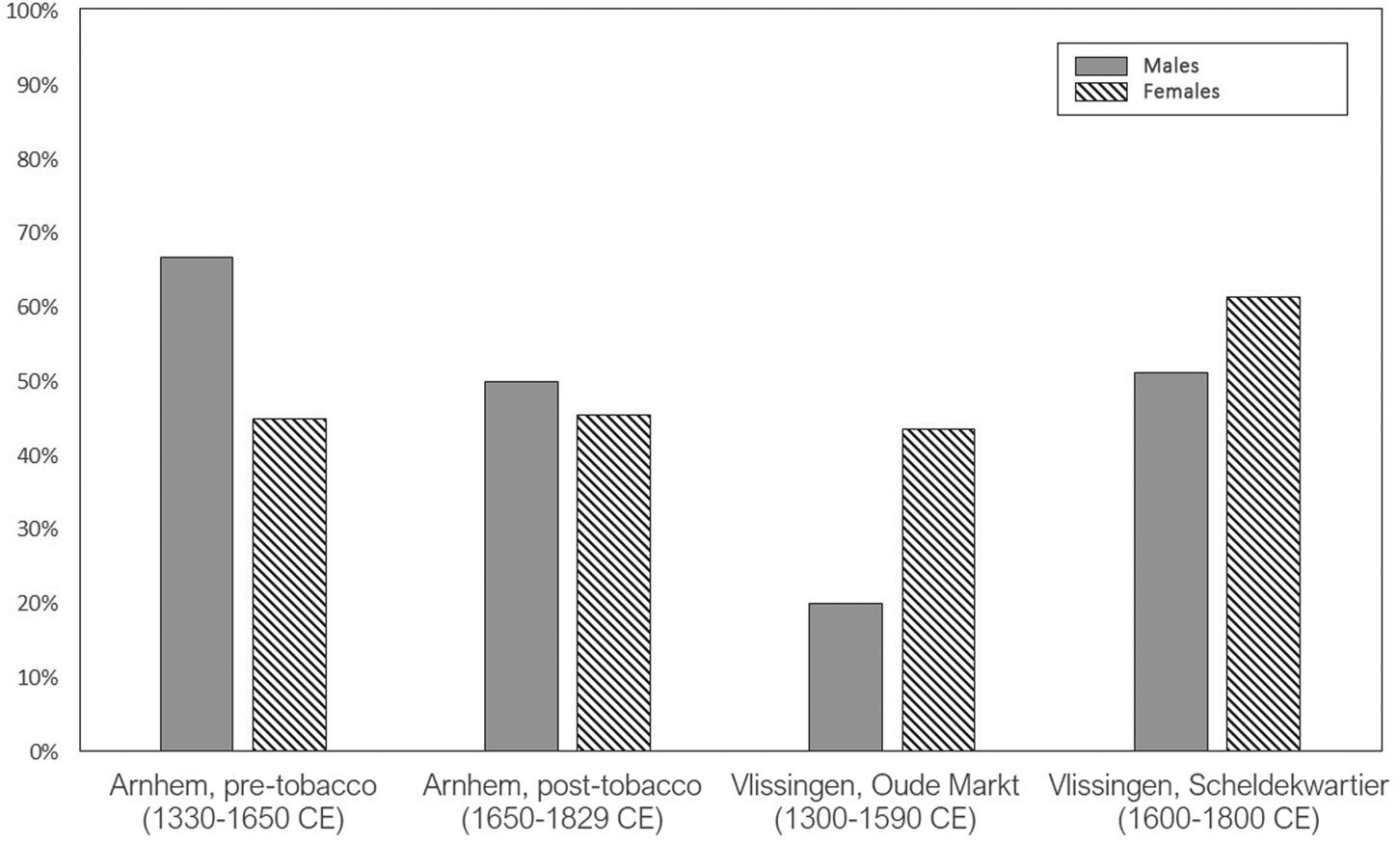
Prevalence of evidence of tobacco consumption among all samples under study, divided by skeletal sex.

Table 4 presents the prevalence rates of tobacco consumption divided by skeletal sex within the samples under study, along with the results of statistical analysis examining their relationship. When investigating tobacco consumption in relation to skeletal sex, we observed that overall evidence of tobacco consumption occurred more frequently in males than females for both post-tobacco sites (Fig. 4). However, statistical tests revealed no significant correlation between skeletal sex and tobacco consumption, pipe notch, and lingua staining in any of the samples under study. In the majority of cases, both pipe notches and lingual staining exhibited relatively even distribution across age-at-death categories for both skeletal sexes (Table 5). Notably, their prevalence appeared to be higher among young and middle adults (20-49 years) compared to older adults (50+ years).

**TABLE 4:** Prevalence rates and statistical analysis of tobacco consumption and skeletal sex in the sample under study.

Comparison	Skeletal assemblage		N	*N* (%)	χ2	*p*-value
Overall evidence of smoking versus sex in Arnhem	Pre-tobacco	Males	42	4 (9.5)	n/a	†0.369
		Females	35	1 (2.9)		
	Post-tobacco	Males	29	17 (58.6)	4.765	0.029
		Females	35	11 (31.4)		
Overall evidence of smoking versus sex in Vlissingen	Oude Markt	Males	16	2 (12.5)	n/a	†1.000
		Females	16	3 (18.8)		
	Scheldekwartier	Males	36	15 (41.7)	1.173	0.279
		Females	28	8 (28.6)		
Evidence of smoking versus sex (*continued*)						
**Skeletal assemblage**			**N**	***N* (%)**	**χ2**	***p*-value**
Arnhem (pre- and post-tobacco)	Pipe notch versus sex	Males	65	16 (24.6)	4.944	0.026
		Females	62	6 (9.7)		
	Dental staining versus sex	Males	71	16 (22.5)	2.379	0.123
		Females	71	9 (12.7)		
Vlissingen (pre- and post-tobacco)	Pipe notch versus sex	Males	46	11 (23.9)	2.724	0.099
		Females	36	4 (11.1)		
	Dental staining versus sex	Males	47	14 (29.8)	0.544	0.461
		Females	36	8 (22.2)		

N = total of individuals with observable feature; *N*= total of individuals presenting pathological lesion; †=Fisher’s Exact Test. Statistical significance was set at *p* = 0.008 according to the Bonferroni correction method

**TABLE 5:** Distribution of dental evidence for smoking (i.e. pipe notch and dental staining) within demographics by skeletal sex and age-at-death groups.

Pipe notch				
		Young adult (20-34 years) (N)	Middle adult (35-49 years) (N)	Old adult (50+ years) (N)
Arnhem, pre-tobacco (1330-1650 CE)	Males	1 (15)	0 (14)	1 (10)
	Females	0 (17)	0 (11)	0 (3)
Arnhem, post-tobacco (1650-1829 CE)	Males	9 (14)	5 (9)	0 (3)
	Females	2 (17)	3 (10)	1 (4)
Vlissingen, Oude Markt (1300-1590 CE)	Males	0 (11)	0 (1)	0 (0)
	Females	0 (7)	0 (2)	0 (1)
Vlissingen, Scheldekwartier (1600-1800 CE)	Males	6 (27)	5 (7)	1 (1)
	Females	2 (21)	2 (4)	0 (1)
Dental staining				
		**Young adult (20-34 years) (N)**	**Middle adult (35-49 years) (N)**	**Old adult (50+ years) (N)**
Arnhem, pre-tobacco (1330-1650 CE)	Males	2 (18)	0 (15)	1 (10)
	Females	1 (19)	0 (14)	0 (3)
Arnhem, post-tobacco (1650-1829 CE)	Males	9 (13)	4 (9)	0 (6)
	Females	3 (19)	4 (11)	1 (5)
Vlissingen, Oude Markt (1300-1590 CE)	Males	1 (13)	1 (3)	0 (0)
	Females	2 (10)	2 (6)	0 (1)
Vlissingen, Scheldekwartier (1600-1800 CE)	Males	9 (28)	3 (6)	1 (1)
	Females	4 (22)	3 (5)	0 (1)

N = total of individuals with observable feature.

## DISCUSSION

According to historical sources, tobacco was first introduced into Europe in the sixteenth century, irrevocably impacting and shaping human society and history (Brongers 1964b; Goodman 2005). Despite the numerous studies that have been published on the history of tobacco in the Netherlands, there seems to be a prevailing narrative based on both historical sources and material culture (e.g. clay tobacco pipes) that: 1) in the Netherlands, tobacco only became truly popular during the course of seventeenth century; and 2) tobacco consumption was predominantly a male habit (Brongers 1964b; Duco 1981; Goodman 2005). However, while the notion of a singular narrative regarding the spread of tobacco across Europe is prevailing, our findings underscore the remarkable variation that characterized this diffusion, emphasizing the need to acknowledge the diverse contextual factors that influenced its adoption and prevalence in different areas of Europe.

Our first hypothesis was that our ‘post-tobacco’ populations (i.e. dating from 1600 CE onwards) would exhibit higher rates of oral indicators of tobacco consumption. Our results suggest that tobacco was popular in Arnhem between 1650-1829 CE. This fits with the overall narrative that tobacco smoking became a common habit by 1630 CE, which is evidenced by 43.7% smoking prevalence rate in the Arnhem, post-tobacco sample. However, we did not observe statistically significant differences in evidence of tobacco consumption in Vlissingen between Oude Markt (1300-1590 CE) and Scheldekwartier (1600-1800 CE). According to historical sources, the cultivation of tobacco was first mentioned in Zeeland in 1610 CE by a local physician (Enthoven 1996). A study on more than 1000 datable pipe fragments recovered from Vlissingen further proposed that tobacco was first introduced in Vlissingen around 1630 CE (Claeys, Jaspers, and Ostkamp 2010). This suggests that tobacco consumption using pipes may not have been prevalent before this date. Our data on pipe notches fully supports this interpretation, as no pipe notches were recorded in individuals dating prior to 1600 CE. However, our data attest that non-pipe-related tobacco use may have been present in Vlissingen well before 1600 CE, indicated by the presence of dental staining, suggesting that tobacco was indeed already a commonly used intoxicant before the introduction of clay pipes. This is especially interesting because, according to Snelders (2021), the first European tobacco users were likely seamen who used to roll dried tobacco leaves in a palm leaf and set it on fire to smoke it. During the 16th and 17th centuries, Vlissingen (and Zeeland in general) had strong trade relations with the Amazon region, where tobacco grew naturally (van Cruyningen 2012). Furthermore, smoking through dried leaves (e.g. cigars) would not cause any dental abrasion (and, therefore, pipe notches), but would likely significantly stain dental surfaces (Dalrymple et al. 2021; Kiliçarslan et al. 2022; Ness, Rosekrans, and Welford 1977). Our results align with this account, as we did not find any significant variation in dental staining across time in Vlissingen. While it must be noted that dental staining may be impacted by various factors such as the consumption of other substances that may cause dental discoloration (e.g. coffee, tea), the presence of dental staining was strongly associated with the presence of pipe notches in three of the skeletal populations we analysed, providing strong evidence that these two markers are indicative of similar processes (i.e. tobacco consumption). We argue that, while tobacco consumption in Arnhem fits the main historical narrative of a rise in popularity around 1630 CE, our results support an earlier spread of tobacco in Vlissingen.

As tobacco has been historically considered a male habit, our second hypothesis was that we would observe higher rates of evidence for tobacco consumption in males than in females. As noted by Snelders (2021) and Brongers (1964b), in the post-medieval period female tobacco use was often associated with immoral behaviour. For example, smelling of tobacco was presented as making women (but not men) sexually unattractive, and the presence of women in public spaces where tobacco was consumed was generally associated with prostitution (Snelders 2021). While it is true that we observed generally higher rates of smoking in males than females, we did not observe any statistically-significant differences between the two skeletal sex groups, suggesting that female individuals were in fact consuming tobacco to a reasonable extent. Similar British historical narratives around tobacco consumption as a predominantly male habit have also been challenged by the findings of Davies-Barrett and Inskip (2024), where evidence of tobacco consumption were observed on 41.0% of female individuals and on 57.6% of males. The discrepancy between our findings and historical portrayals of female smokers carries deep implications for understanding female tobacco consumption in the past. A potential explanation for this discrepancy is that historical representations of female smokers as linked to poverty and/or prostitution may have originated from predominantly male perspectives aiming to define and regulate what they perceived as a male activity (McShane 2021; Davies-Barrett and Inskip 2024). The negative societal views surrounding female smoking may have compelled women to consume tobacco differently from men. While male smoking was often a social activity conducted in public spaces (Snelders 2021), female tobacco consumption was likely a more private endeavour, enjoyed within the confines of private homes or with other women (McShane 2021). The societal pressure against women smoking may also explain the relatively low occurrence of female pipe notches we observed in our samples. Unlike men, who were likely quite accustomed to pipe smoking from young ages, women might have opted for less conspicuous methods such as chewing tobacco leaves or using less abrasive mouthpieces, which would have caused less noticeable damage to their teeth (Dekker 2013). While our study did not reveal any statistically significant correlation between skeletal sex and a specific marker of tobacco consumption (i.e. notches/dental staining), it is essential to acknowledge that the limitations of our sample size may have impacted our observations. Further investigation into the distribution of these markers among different skeletal sex categories could offer fresh insights.

While our overall sample size was adequate for the purpose of this research, its breakdown into skeletal sex and age-at-death categories revealed some limitations in interpreting our results. Specifically, we observed that skeletal markers for tobacco consumption were more prevalent among young and middle adults compared to older adults. Given the permanent nature of both pipe notches and dental stains without surgical intervention, this observation prompts speculation that smokers may have experienced higher mortality rates than non-smokers. However, our ability to draw definitive conclusions about observed prevalence rates is hindered by the disproportionately lower number of old adults with observable features compared to other age-at-death categories. Addressing this challenge remains complex, as it is inherent to the nature of osteoarchaeological research. Furthermore, the accurate osteoarchaeological identification of individuals who engaged in smoking has to-date its limitations, and evidence for other forms of tobacco consumption (e.g. chewing, snuff taking) have yet to be identified in human osteological remains. Consequently, it is plausible that the prevalence of smokers within our sample surpasses our observations. Moreover, although there appears to be a significant link between the existence of pipe-notches and dental staining (Walker and Henderson 2010), it is crucial to acknowledge that the exact mechanism behind the formation of any type of dental staining remains today poorly comprehended both clinically and archaeologically. Further advancements in enhancing our understanding of the mechanisms and attributes underlying dental lingual staining is essential. Moreover, the advancement of future biomolecular analyses (Badillo-Sanchez et al. 2023; Bartholdy et al. 2024) may provide the means to further explore tobacco consumption among different societal groups.

Another limitation was posed by the dating of our samples. While historical records emphasize a pivotal 40-year period spanning from 1590 to 1630 CE for the dissemination of tobacco among Dutch populations, our osteoarchaeological investigations encountered limitations in precisely identifying individuals from this timeframe within our samples. Consequently, in the Arnhem sample, this period fell within our ‘pre-tobacco’ sample. Our osteological findings support the hypothesis that tobacco was not prevalent in Arnhem before 1650 CE. However, revisiting this analysis using a skeletal sample exclusively from the period 1590-1630 CE holds promise for shedding new light on the spread of tobacco consumption within Dutch societies.

Finally, more research is needed to investigate the many ways tobacco has shaped and influenced our society and culture. For example, larger sample sizes may allow for a better understanding of how social groups that have received less attention, such as women and children, were involved not only in the smoking practice, but also in the tobacco industry (Benedetti et al. 2021). The inclusion of more diverse skeletal collections may also allow a better understanding of how tobacco was not only ‘popular among all socioeconomic classes’ but how its consumption changed on the basis of wealth and societal roles, among other factors (Brongers 1964b; Davies-Barrett and Inskip 2024). As more research into the complexities of tobacco in the Netherlands is underway, we stand to gain valuable knowledge about past societies, shedding light on cultural practices, social interactions, and historical trends.

## CONCLUSIONS

By examining a large sample of skeletal remains, this paper presents a comprehensive investigation into the historical spread and impact of tobacco consumption in the Netherlands, with a particular focus on the urban centres of Arnhem and Vlissingen. While historical sources and material culture have contributed to a prevailing narrative on tobacco’s history, the findings from this study challenge and enrich our understanding of this complex phenomenon.

Contrary to the prevailing notion that tobacco became popular in the Netherlands only around 1630 CE, our results suggest that its popularity in the region of Vlissingen may have begun much earlier with tobacco chewing and/or cigars, potentially evolving into pipe smoking practices later on. Similarly, while historical records and previous studies have focused on the prevalence of tobacco consumption as a male habit, our research reveals that women also likely consumed tobacco to a significant extent, underscoring the importance of re-considering gender-specific assumptions from historical sources.

In conclusion, our paper has made significant contributions to the history of tobacco consumption in the Netherlands by challenging prevailing narratives with empirical osteological data. By presenting nuanced findings, our study encourages new research to embrace the contextuality of tobacco’s diffusion and its impact on various societal and historical aspects. Future research into the archaeology of tobacco consumption in the Netherlands will undoubtedly benefit from expanding to a wider and more diverse sample of human skeletal remains, for which information such as socioeconomic class and/or occupational activities is known.

## DATA DEPOSITION

The datasets generated and analysed during the current study are available at Casna, M., 2023, ‘Data for: “Smoking histories”’, https://doi.org/10.17026/AR/R1Y1KM, DANS Data Station Archaeology.

## Note

1 According to the Paleoanthropology Dental Notation, the upper teeth are denoted I^1^, I^2^, C, Pm^3^, Pm^4^, M^1^, M^2^, and M^3^. The lower teeth are: I_1_, I_2_, C, Pm_3_, Pm_4_, M_1_, M_2_, and M_3_. Left or right is specified by the first letter (i.e., L, R) (see Scott and Turner 1997).
